# Investigating rare and ultrarare epilepsy syndromes with *Drosophila* models

**DOI:** 10.12703/r/10-10

**Published:** 2021-01-29

**Authors:** Paul Lasko, Kevin Lüthy

**Affiliations:** 1 Department of Human Genetics, Radboud University Medical Centre, Nijmegen, Netherlands; 2 Department of Biology, McGill University, Montréal, Québec, Canada; 3 Donders Institute for Brain, Cognition and Behaviour, Radboud University Medical Centre, Nijmegen, Netherlands

**Keywords:** Neurological disorders, Epilepsy, Drosophila, Neurogenetics, Disease model

## Abstract

One in three epilepsy cases is drug resistant, and seizures often begin in infancy, when they are life-threatening and when therapeutic options are highly limited. An important tool for prioritizing and validating genes associated with epileptic conditions, which is suitable for large-scale screening, is disease modeling in *Drosophila*. Approximately two-thirds of disease genes are conserved in *Drosophila*, and gene-specific fly models exhibit behavioral changes that are related to symptoms of epilepsy. Models are based on behavior readouts, seizure-like attacks and paralysis following stimulation, and neuronal, cell-biological readouts that are in the majority based on changes in nerve cell activity or morphology. In this review, we focus on behavioral phenotypes. Importantly, *Drosophila* modeling is independent of, and complementary to, other approaches that are computational and based on systems analysis. The large number of known epilepsy-associated gene variants indicates a need for efficient research strategies. We will discuss the status quo of epilepsy disease modelling in *Drosophila* and describe promising steps towards the development of new drugs to reduce seizure rates and alleviate other epileptic symptoms.

## Introduction

### Diversity of epilepsy genetics

Epilepsy has a strong hereditary component; over the past decade, more than 100 causative epilepsy genes have been discovered by worldwide epilepsy screening consortia^1,2^. Approximately 50% of cases can be traced to single genetic mutations^2–4^. The identification of these genes by worldwide consortia is an important step toward the development of new therapeutic approaches because modeling the disease conditions requires a powerful genetic approach. Given the rapid progress in stem cell technology, patient-derived models are one attractive avenue of investigation^5^. However, the heterogeneity of human genomes poses challenges, as differences in genetic background can alter the phenotype produced by a given mutation. These challenges can be partially overcome in the *Drosophila* model through crossings to achieve isogenized lines, as well as the standardization of transgenic insertions to reach a stable genetic background^6–9^. However, genetic drift remains a concern when transgenic lines are kept over many generations and can lead to differing results from different labs that ostensibly use the same stocks.

Anti-seizure drugs (ASDs) aim to control epileptic symptoms and improve quality of life in epilepsy patients. Despite very good results for two-thirds of those affected, seizures remain refractory, or drug-resistant, in one-third of patients^10^. Even increased dosage or combination therapy has not produced a significant reduction in the fraction of intractable cases. In addition, epilepsy patients can develop further comorbidities despite seizure suppression, including intellectual disability (ID) and autism spectrum disorder^11,12^.

### Artificial intelligence-based detection of epilepsy-associated phenotypes

**Figure 1.**
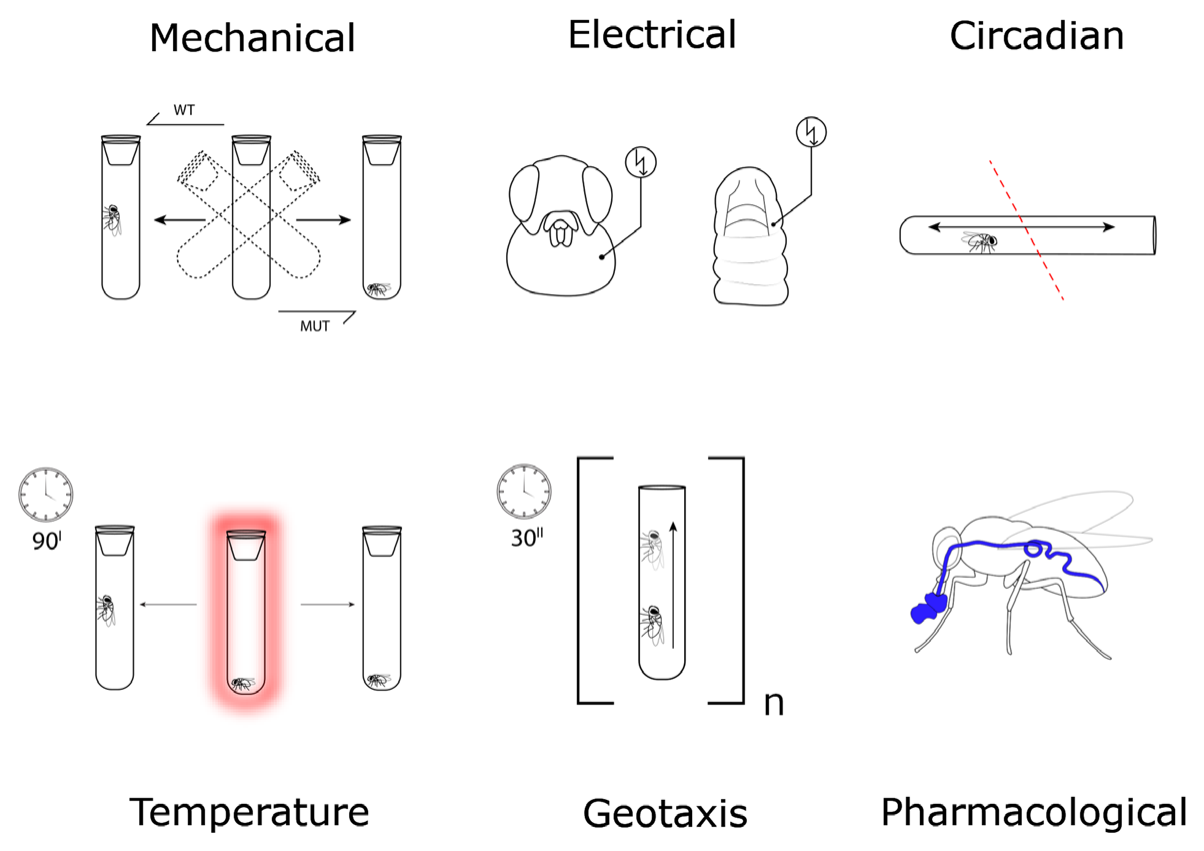
Epilepsy models utilizing *Drosophila* behavior. Stimulation of *Drosophila* can induce seizure-like behavior in susceptible animals. Quantification typically reflects the recovery after stimulation, the frequency and duration of seizuring effects, or significant behavioral changes. Circadian rhythm (sleep/wake) and geotaxis assays measure the behavior of flies in a test chamber to reveal susceptibility to either seizure-like or complex behavior changes associated with epileptic pathology. The paradigms can be combined with typically oral uptake of pharmacological compounds to induce or suppress these phenotypes. MUT, mutant; WT, wild type.

To what extent can behavior testing be automated in data analysis? Several readouts have been developed, including the tracking of fly movements by video analysis to capture either momentary adult fly seizure-like behavior or long-term movement and activity patterns^13–15^. Readouts of larval motor coordination have also been employed in seizure-associated studies^16^. An intriguing alternative to screens searching for genetic modifiers is the Janelia Fly Olympiad project. In this project, 2,205 specific anatomical regions were acutely silenced using *shi^ts^* or overexcited using *TrpA1* in order to track behavior changes automatically utilizing computer vision algorithms^17,18^. This technical approach registered a broad range of behavior patterns, including locomotion, coordination, and climbing that together reflect epilepsy-associated paradigms^19^. Righting behavior and side-steps, for instance, have been previously employed to quantify epilepsy-associated phenotypes^20,21^. The combination of increasingly efficient behavior readouts and genetic screening libraries enabling the regulation of nearly all genes with tissue-specific promotors holds great promise for future screens, in which the advantages and limitations of the chosen readout (Figure 1) will determine the successful identification of disease-relevant phenotypes. This promises to extend our knowledge beyond the identification of single genes toward learning about how they interact to produce disease states.

### Disease modeling in *Drosophila* and screens for conserved, disease-associated factors

The fruit fly *Drosophila melanogaster* has contributed a great deal to genetic research, and around 70% of the fly’s genes have orthologs in humans^22^. Through experiments ranging from ethological to molecular biological assays, key insights have been gained from *Drosophila* into the genetic basis of hereditary disorders, including Alzheimer’s disease and epilepsy. Forward genetic screens in *Drosophila* have identified factors affecting general functions such as sleep^23^ as well as many that associate with ID and autism spectrum disorder^24^. With respect to epilepsy, geneticists and clinicians have identified causative variants in more than 100 genes, the majority of which have conserved orthologs in *Drosophila*, enabling fast and inexpensive modeling of human patients’ conditions^25,26^. Even the intricate role of glial cell contribution to hereditary epilepsies has been investigated through interactions that have been conserved on the level of both molecular and cellular interactions^27,28^. The important contribution of *Drosophila* to epilepsy research is reflected in a Google Scholar search for <‘*Drosophila*’ AND ‘epilepsy’> that yields 28,200 publications, including around 12,000 from the past 5 years, as well as 700 related patents.

Here, we outline the current state of epilepsy modeling in *Drosophila* and indicate methodological innovations that enable new approaches to disease modeling, such as CRISPR–Cas9 genetic screens.

## Epilepsy modeling in *Drosophila*

### Behavior experiments

*Drosophila* has been used to model epilepsy for nearly 50 years, since the discovery of bang-sensitive alleles^29^. These genetic mutations cause the animals to respond to a mechanical or temperature stress with a characteristic, seizure-like response. Three criteria for epilepsy model readouts have been established, namely quantifiable seizure susceptibility thresholds, a baseline genetic control, and resemblance to human seizuring^30^.

The fly model helped to determine that seizure-like behavior can be evoked by global central nervous system activity that, at least for the characteristic muscle tremors, requires long-range interactions in the nervous system rather than regionally compartmentalized foci of activity^21^. While global brain excitation is sufficient to induce an attack, in classic bang-sensitive alleles only the involvement of certain brain regions was necessary in order to elicit bang sensitivity or temperature sensitivity. These epilepsy paradigms required the genetic targeting of different, yet overlapping, brain regions in order to initiate the respective seizure-like effects, providing an insight into the complexity of invertebrate analogous behavior to epileptic seizuring (Table 1).

**Table 1.** Well-characterized seizure-associated genes in *Drosophila*.

Gene name	Homolog	Bang	Heat	Unc.	Function	Reference
*Paralytic (para)*	SCN1A	+	+	+	Na^+^ channel α subunit	Parker *et al*., 2011^31^
*slowpoke (slo)*	KCNMA1	-	+	-	K^+^ channel subunit	Moss *et al*., 1996^40^
*seizure (sei)*	KCNH2/7	+	+	+	K^+^ channel subunit	Titus *et al*., 1997^41^; Wang *et al*., 1997^42^
*easily shocked (eas)*	ETNK1	+	-	-	Ethanolamine kinase	Pavlidis *et al*., 1994^43^
*Shaker (Sh)*	KCNA1	-	-	+	K^+^ channel α subunit	Salkoff & Wyman, 1981^44^
*mustard (mtd)*	OXR1	+	-	-	Oxidative stress sensor	Wang *et al*., 2019^45^
*shaker B (shakB)*	-	-	+	+	Gap junction subunit	Crompton *et al*., 1995^37^

The closest human ortholog and experimental results for bang sensitivity, heat sensitivity, and uncoordinated behavior are listed. The bang sensitivity negative mutant flies displayed neurophysiological or motor defects prior to stimulation.

Next, we elaborate on these respective assay types and summarize behavioral and physiological assays most relevant to epilepsy-associated behavior (Figure 1) in the context of representative studies.

***Mechanical stimulus.*** The** discovery of mutations in genes coding for sodium and potassium channels led to mechanistic insights into neuronal excitability, e.g. *Drosophila* mutants with corresponding human orthologs *paralytic*/SCN1A, *slowpoke*/KCNMA1, and *seizure*/KCNH2/KCNH7^31^. In parallel, the orthologous human epilepsy variants were found to occur with high prevalence in patients^32^. It is important to stress that bang sensitivity describes a specific, seizure-like animal behavior pattern as opposed to general loss of activity. This behavior pattern begins once a threshold has been reached and occurs in an all-or-nothing manner through a periodic sequence of shaking and loss of coordination. The inactive phase has been found to be disrupted by a tap to the experimental chamber, indicating that the internal state of the central nervous system in seizure-like attacks is directly affected by external stimuli.

Mechanical induction of seizure-like states has identified alleles in more than 50 genes, again including orthologs of causative disease genes^21,29,33^. In some cases, epilepsy-related phenotypes were revealed through refined genetic strategies since full knockout alleles can result in adult-lethal animals^34–36^.

A functionally related group of gap junction genes has been identified owing to the characteristic induced shaking, beginning with *shaking B* (*shakB*) and its interaction with *ogre*/Innexin1, but also *stress-sensitive B* (*sesB*)^37–39^. Despite substantial divergence in these genes between human and *Drosophila*, their role in human seizure progression and in blocking seizures means that the insights from the *Drosophila* model nevertheless hold translational value^46^.

A further 155 genes are tagged as *uncoordinated* and 42 more are linked to further epilepsy-relevant readouts in the FlyBase Human Disease Model Report (flybase.org/lists/FBhh, Table 1). These represent an understudied set of potential epilepsy models.

***Temperature elevation.*** Mutations in 1,200 *Drosophila* genes have been registered as associated with heat sensitivity. Increasing the temperature of ectothermal animals increases the temperature of the central nervous system and induces the stereotypic seizure response in sensitized genetic models^21,33–35,47^. Surprisingly few (~30%) of the genes that can mutate to produce mechanically induced phenotypes co-occur with those that are temperature dependent. This is consistent with findings in the *paralytic* (*para*) mutant *para^bss1^*, which demonstrated that, in spite of effects on global brain activity underlying responses to mechanical or thermal stress, the effects are linked to distinct brain regions^21^. While a strong focus in behavioral experiments has been placed on activity bursts resembling seizures, other approaches attempt to capture paroxysmal epileptic dystonia or paralysis^33,48^.

***Electroconvulsive stimulus.*** Detection of such non-seizuring events extends the range of epilepsy modeling in *Drosophila* and has benefited most from electrophysiological readouts^30^. Hyperexcitation in the central nervous system was induced through short electroconvulsive electric pulses lasting 0.1 to 0.5 milliseconds up to 3 second pulses^49–51^. Electric stimulation at larval stages allows investigation of adult-lethal seizure alleles in *Drosophila* such as *slamdance* (*sda*), as it induces characteristic tremors in mutants that are visible in the high-contrast mouth hooks that can be seen in the otherwise translucent body^49–51^. The paradigms detailed in this and the previous two sections elicit heightened brain activity from where the giant fiber system plays an important role conveying stereotypic, seizure-like motor responses to the motor system, with the interesting exception of flight muscle^49^. This commonality suggests a degree of convergence in generating epilepsy-associated behavior patterns.

***Genetic stimulus.*** Mutants in the *para* locus, such as the *para^bss^* hypermorph allele as well as knockdown alleles (as discussed in ‘Tissue-specific genetic screening’ below), have been thoroughly investigated for seizure-like behavior^52^. The alpha subunit of voltage-gated sodium channels Para mediates neuronal action potentials. The human orthologs *SCN8A*, *SCN2A*, and *SCN1A* are strongly associated with pathogenic epilepsy phenotypes and carry several of the most frequent alleles associated with epilepsy^53–55^. *Drosophila* carrying the *para^bss^* mutation are sensitized to induced seizure-like attacks, notably even through optogenetic stimuli^31,56^. *para^bss^* mutants have also received attention for the identification of genetic interactors that suppress seizuring, among them *gilgamesh* (*gish*), which encodes a casein kinase 1 that is highly conserved in humans^57^. Genetic seizure modifier genes and downstream interacting genes are efficiently identified in *Drosophila*. An alternative case of predisposition for seizure-like behavior is the metabolic epilepsy model for *PNPO* deficiency (OMIM #610090). Induction through a sugar-only diet in mutants of the fly gene *sugarlethal* (*sgll*), the orthologue of *PNPO*, elicited seizures without additional acute stimuli^58^.

Some bang-sensitive alleles are temperature sensitive, with *shibire^ts^* (*shi^ts^*) the most prominent example. *shi* encodes a dynamin and one of its human orthologs, *DNM1*, is linked to an early infantile epileptic encephalopathy (OMIM #616346). These alleles produce proteins that lose function at conditions warmer than room temperature. Dynamin’s functional relevance for epilepsy modeling was demonstrated by suppressing the seizure-like phenotypes of the well-known bang-sensitive genes *para*, *easily shocked* (*eas*), and *sda* through heat activation of the *shi^ts^* allele^59^. A further genetic rescue is the suppression of seizures in the *TBC1D24* epilepsy and DOORS Syndrome (OMIM #220500) ortholog *skywalker* (*sky*), which was mediated by the *shi* effector *Synaptojanin* (*Synj*)^34^.

***Circadian rhythm.*** An environmental stimulus is required to induce seizure-like behavior in *Drosophila* carrying genetic mutations, with rare exceptions such as the *prickle* mutant *pk^spl^* or the *zydeco* mutant *zyd^1^* when coupled with one out of several *repo>dStim* knockdown lines^60,61^. One potential way to observe behavioral changes without triggering an acute response is to monitor sleep or activity patterns, and mutants that have seizure-like phenotypes often also have abnormal sleep. For example, shorter or more fragmented sleep periods were observed in the uncoordinated mutant *Shaker* (*Sh*)^62^, which is homologous to the epilepsy gene *KCNA2* (OMIM #616366), and for *sky*^34^. Several other well-studied bang sensitivity alleles are also sleep defective, including those affecting the epilepsy gene orthologs *I_h_ channel* (*Ih*), *para*, and *shi*, as well as two direct interactors of *Sh* potassium channels, *quiver* (*qvr*), and *Shaker cognate b* (*Shab*)^63,64^. This link between sleep abnormalities and seizures requires further investigation in humans and in animal models to better understand its applicability in clinical epilepsy research.

## Model-based steps towards effective epilepsy treatment

The identification of over 100 rare genetic epilepsies suggests a broad range of disease mechanisms, such as synaptic transmission defects, channelopathies, and transcriptional, metabolic, and transport defects^3,65–67^. Despite this progress, most underlying mechanisms leading to epileptic diseases remain to be elucidated^4^. Systematic classification of causative genes is currently based on phenotypic patient descriptions. Genetic trends in the age of onset were determined through phenotypic distinction of 12 different groups, while at the same time a major overlap of the genotype–phenotype spectrum became apparent^48^. Mutations in genes coding for ion channel subunits underlie a high number of patient cases among hereditary epilepsies^68^. An example are causative variants in *SCN1A* which are the most frequent cause of Dravet syndrome: more than 80% of patients carry variants of this ion channel subunit and are in general highly resistant to treatment^69^.****

To identify potential new treatments for Dravet syndrome and further drug-resistant conditions effectively, a high-throughput *in vivo* model can reveal brain-specific genetic interactors involved in epilepsy. Screens for suppressors of seizure-inducing alleles can reveal novel drug targets and point to related functional pathways. Previous large screens have identified genetic suppressors of seizure-like phenotypes. Alleles disrupting neuronal communication were also identified in non-ion channel genes, e.g. *eas* mutant seizure-like behavior or *sky*/*TBC1D24* and *synj*/*SYNJ1*. In fact, the link in *Drosophila* between *synj* and epilepsy was found before human patient variants in *SYNJ1*-causing untreatable epilepsy had been identified^70–72^. Beyond screening for phenotypical resemblance to seizures, suppressor screens, for instance in the genetic background of *para^bss^* mutant animals, determined genetic interactors of epilepsy-associated genes^31,57^. Suppressors have been identified in both unbiased and selective genetic screens, e.g. for *sky*/*TBC1D24*^34,71,73^.

A large inhibitory RNA (RNAi)-based screen for behavioral phenotypes resulting from gene inactivation in glial cells has identified a number of bang-sensitive mutants, which are orthologs of human epilepsy-associated genes with roles in glial and, interestingly, glia–neuron interactions^16,74,75^.

In addition to mutant and knockdown models, the *Drosophila* community has developed 192 genetically inbred, or isogenized, lines known as the *Drosophila melanogaster* Genetic Reference Panel (DGRP), which enable the connection of epilepsy-associated phenotypes with multiple endogenous genetic loci, potentially leading to the discovery of new genetic modifiers^76^.

### Tissue-specific genetic screening

Comprehensive libraries of transgenic lines are facilitating epilepsy and further genetic screens, among them very prominent collections of RNAi lines covering 91% of *Drosophila* protein-coding genes^77^. These genetic libraries by the Vienna *Drosophila* Resource Center and the Transgenic RNAi Project at Harvard Medical School have the advantage of tissue-specific knockdown, whereas knockout lines, such as the MiMIC insertions which introduce insertion cassettes, often constitutively abrogate gene expression^78,79^. The RNAi-based screens are as versatile as the promotor lines combined with transcript knockdown, yet their effect strength has been found to vary and should be assessed independently from the phenotype^80^. Another 10,000 fly lines enabling knockdown in different neuronal and glial subsets have been generated in the Fly Light project^81^. Similar resources that take advantage of the CRISPR–Cas9 gene targeting system are under development (see Box 1).

**Box 1. CRISPR–Cas9-based knockouts in screening**

Inhibitory RNA (RNAi) knockdowns, while highly useful, are variable in the degree to which they inactivate the target gene. Libraries of fly stocks that express Cas9 and UAS-driven short-guide RNA elements will enable tissue-specific complete knockouts of any targeted gene^82^. These libraries will be a valuable alternative to the RNAi-based resources that are presently in broad use.

### Drug screens utilizing *Drosophila*

The development of epilepsy treatments in animal models focuses on seizure-like readouts that can be suppressed through genetic interaction or application of pharmacological compounds^83,84^. Akin to genetic interactor screens, the interplay of seizure phenotypes with pharmacological inhibitors has been investigated, based on both known ASD as well as on novel substances. Picrotoxin-induced seizuring or genetic models such as *eas* flies served as screening models^33,49,52,85^. Testing known ASDs discovered responsiveness to pharmacological inhibitors such as phenytoin and nifedipine, but also to gabapentin, compounds that are effective against severe seizure pathologies^84,85^. Just as in human epilepsies, screens found no single drug compound which could effectively suppress the many different underlying genetic changes in *Drosophila*^11^.

One potential limitation of focusing on seizure-like behavior readouts in the fly model is the omission of the wider phenotypic spectrum in epileptic encephalopathies, regarding both the method of treatment and its timing^86,87^. ID and autism spectrum disorders are examples of strongly co-occurring pathologies which are beginning to be integrated into current epilepsy and neurodevelopmental disease models in small animal models or in drug trials^12,88–90^.

### Future perspectives in drug screening

Patients with drug-resistant epilepsy have a restricted quality of life and higher mortality, and the drug resistance may have cognitive and psychosocial consequences^11^. ASD alternatives are surgical intervention, neurostimulation, ketogenic diet, and lifestyle adjustment. Recent clinical studies suggested cannabidiol-based treatment as a therapeutic intervention in drug-resistant therapies and successful seizure suppressors in a *Drosophila* model^91,92^. Despite the enormous amount of research and the increasing number of available ASDs, there still remain epileptic patients who cannot be treated^11^.

### Unraveling epilepsies from two ends: patient variants or functional pathways

The number of epilepsy-associated genes was expanded drastically when a whole-exome study of more than 17,000 individuals detected vast numbers of ultra-rare variants^4^. The large number of underlying genes combined with the small patient groups affected by individual variants require inexpensive and rapid methodologies to test anti-seizure measures. As discussed above, while stem cell-based methods potentially reveal precision medicine solutions, at the current moment *Drosophila* genetic screening offers rapid and economical insight into the genetic function and interactome of conserved genetic variants.

In a complementary direction, unbiased study of molecular pathways and genetic interactions connects known genes with each other as well as with new genes or unexpected causative variants in epilepsy. For example, *mtd/OXR1* and *sky/TBC1D24* variants cause epilepsies that were found to be linked to reactive oxygen species levels and modified by anti-oxidants at the level of neurons as well as the entire brain, based on humanized *Drosophila* transgenic models in which the cDNA of patient variant *OXR1* or *TBC1D24* was expressed in the brain^45,48^. Humanization of the most frequent genetic cause underlying Dravet syndrome, point mutations in *SCN1A*, have enabled detailed studies of the effect conveyed by many of the mutations on protein function^93^. By understanding the mechanistic and functional connections between epilepsy-associated genes, a step from many unconnected single disorders to functional networks becomes more tangible. A third way to assess in which wider gene networks these single factors are functionally required is based on systems biology, specifically on brain transcriptomics^94,95^. Computational approaches have the potential to connect mouse and human *in vitro* data with *Drosophila* epilepsy *in vivo* models to obtain integrated conserved functional networks. This approach will hold validity across a range of disorders affecting the central nervous system^7,8^.

## Future perspective

Epilepsy patient genetics provide unprecedented insights into common and more rare epilepsy conditions, many resistant to current treatment forms. Beyond their clinical phenotype, a shared genetic basis has been revealed that allows the investigation of converging genetic disease mechanisms^96^. Based on epilepsy genes identified by the International League Against Epilepsy (ILAE), previous computational approaches have been validated in cellular and mouse models, leading to a proposed mechanism of valproic acid function in *SCN1A* patient cases and to pre-clinical confirmation of Csf1r inhibitors as novel ASD candidates^94,95^. With the advent of whole-brain single-cell transcriptome datasets, recent developments have enabled the inference of connections between the orthologs of causative monogenic epilepsy factors at unprecedented resolution and will in the future support models of epilepsy gene networks^97,98^.

Grouping disease genes based on inferred genetic interactions and confirming candidate networks in efficient animal screens could become a valuable additional tool for expanding drug classifications of pre-clinical epilepsy models, associating epilepsies on their responsiveness to treatment as a new criterion. Functional connections and ‘epilepsy gene networks’ could guide genetic and pharmacological screens and ultimately future treatment strategies.
